# Retinal vascular and structural changes are associated with amyloid burden in the elderly: ophthalmic biomarkers of preclinical Alzheimer’s disease

**DOI:** 10.1186/s13195-017-0239-9

**Published:** 2017-03-01

**Authors:** S.Mojtaba Golzan, Kathryn Goozee, Dana Georgevsky, Alberto Avolio, Pratishtha Chatterjee, Kaikai Shen, Vivek Gupta, Roger Chung, Greg Savage, Carolyn F. Orr, Ralph N. Martins, Stuart L. Graham

**Affiliations:** 1 Vision Science Group, Graduate School of Health (Orthoptics Discipline), University of Technology Sydney, 15 Broadway, Ultimo, NSW 2007 Australia; 2 Department of Clinical Medicine, Faculty of Medicine and Health Sciences, Macquarie University, Sydney, NSW Australia; 3 KaRa Institute of Neurological Diseases, Macquarie Park, NSW 2113 Australia; 4 Department of Biomedical Sciences, Macquarie University, Sydney, NSW Australia; 5 Anglican Retirement Village, Sydney, NSW Australia; 6 School of Psychiatry and Clinical Neurosciences, University of Western Australia, Perth, WA Australia; 7 Australian e-Health Research Centre, CSIRO Health and Biosecurity, Herston, QLD Australia; 8 ARC Centre of Excellence in Cognition and its Disorders, Department of Psychology, Macquarie University, Sydney, NSW Australia; 9 Macquarie Neurology, Macquarie University, Sydney, NSW Australia

**Keywords:** Retinal imaging, Vascular biomarkers, Alzheimer’s disease, Early diagnosis

## Abstract

**Background:**

Retinal imaging may serve as an alternative approach to monitor brain pathology in Alzheimer’s disease (AD). In this study, we investigated the association between retinal vascular and structural changes and cerebral amyloid-β (Aβ) plaque load in an elderly cohort.

**Methods:**

We studied a total of 101 participants, including 73 elderly subjects (79 ± 5 years, 22 male) with no clinical diagnosis of AD but reporting some subjective memory change and an additional 28 subjects (70 ± 9 years, 16 male) with clinically established AD. Following a complete dilated ocular examination, the amplitude of retinal vascular pulsations and dynamic response, retinal nerve fibre layer thickness and retinal ganglion cell layer (RGCL) thickness were determined in all patients. Systemic blood pressure and carotid-to-femoral pulse wave velocity were measured. The elderly cohort also underwent magnetic resonance imaging and ^18^F-florbetaben (FBB)-positron emission tomographic amyloid imaging to measure neocortical Aβ standardised uptake value ratio (SUVR), and this was used to characterise a ‘preclinical’ group (SUVR >1.4).

**Results:**

The mean FBB neocortical SUVR was 1.35 ± 0.3. The amplitude of retinal venous pulsations correlated negatively with the neocortical Aβ scores (*p* < 0.001), whereas the amplitude of retinal arterial pulsations correlated positively with neocortical Aβ scores (*p* < 0.01). RGCL thickness was significantly lower in the clinical AD group (*p* < 0.05).

**Conclusions:**

The correlation between retinal vascular changes and Aβ plaque load supports the possibility of a vascular component to AD. Dynamic retinal vascular parameters may provide an additional inexpensive tool to aid in the preclinical assessment of AD.

## Background

Alzheimer’s disease (AD) is the most common form of dementia, accounting for 60–70% of all dementia cases. AD is a progressive neurodegenerative disorder causing irreversible deterioration in cognitive function secondary to neuronal cell death and brain atrophy [1]. The main pathological features are the accumulation of two proteins: amyloid-β (Aβ) peptide, generated from its parent molecule, the amyloid precursor protein (APP), which aggregates into extracellular plaques, and hyper-phosphorylated tau, which forms intracellular neurofibrillary tangles [2]. Evidence now implies that neuropathological changes involving the accumulation of Aβ occur up to 20 years prior to the emergence of clinical symptoms, emphasising the importance of developing methods for early diagnosis [3].

Currently, diagnosis is based primarily upon cognitive assessments of patients presenting with symptomatic features of cognitive and behavioural change [4, 5]. On the basis of these assessments, an individual may fall into one of three categories: (1) probable AD dementia, (2) possible AD dementia or (3) probable or possible AD dementia with evidence of the AD pathophysiological process [5]. Computed tomography and/or magnetic resonance imaging (MRI) are frequently used clinically in the initial diagnosis of cases where probable AD is likely, whereas positron emission tomographic (PET) amyloid imaging is less frequently included where confirmation is needed; however, this is not universal [6]. Johnson and colleagues [7] devised a set of criteria that an individual must meet before PET amyloid imaging can be considered ‘appropriate’. These criteria include (1) a cognitive complaint, (2) possible AD diagnosis and (3) whether the knowledge of the presence or absence of Aβ will increase diagnostic accuracy or certainty. However, PET is not applicable for use in population-wide screening, owing to the relatively high cost of this test. Besides PET amyloid imaging, cerebrospinal fluid (CSF) pathophysiological markers, including Aβ_1–42_, total tau and phosphorylated Tau, have also shown high specificity in confirming AD pathophysiology [4].

Because the retina is considered an extension of the central nervous system (CNS) via the optic nerve, researchers have investigated it to determine whether it can serve as a marker for AD change [8]. The advantages of including the retina in investigations for diagnosing probable AD include its low cost, easy accessibility and the non-invasive nature of the tests. Recently, researchers have identified Aβ deposits in eyes from both human AD and transgenic mouse models. Retinal imaging techniques for Aβ are being investigated for detection of potential biomarkers for preclinical AD [9]. Although human studies have identified retinal abnormalities in patients with AD, such as reduced retinal nerve fibre layer (RNFL) thickness [10] and degeneration of retinal ganglion cells [11], these deficits are not AD-specific and are seen in patients with other degenerative diseases, such as glaucoma [12]. Animal models including *APP*/*PS1*-transgenic mice show Aβ plaques depositing in the retina, and these plaques have been correlated with plaque load in the brain [1]. The investigators in the latter study also reported detecting the amyloid burden in the retina at 2.5 months of age, which preceded the brain deposition occurring from 5 months of age.

Researchers have also identified a potential association between changes in vascular parameters, both static and dynamic, and AD changes. These include retinal vessel diameter [13, 14] as well as systemic cardiovascular measurements of blood pressure and pulse wave velocity (PWV) [15, 16]. Amyloid plaques have been reported to be closely located to blood vessels in the brain [17]. Systemic changes in parameters such as increased blood pressure [15] and arterial stiffness [16] have been correlated with declining cognition. In *APP*/*PS1*-transgenic mice, increased blood pressure was associated with higher concentrations of Aβ plaques in the brain. In addition, endothelial cell function includes regulation of APP, which could support the concept of increased mechanical stress as a contributing factor in the overproduction of Aβ in the vasculature.

Therefore, in this study, we aimed to determine if there was an association between retinal vascular parameters and neocortical Aβ concentrations. Vascular changes and PET amyloid scan results were documented in an elderly group of participants who had subjectively reported some memory issues but had no clinical signs of AD confirmed in testing. Vascular changes identified were then also investigated in a known group with clinically diagnosed AD dementia.

## Methods

### Study design

A total of 101 participants were included. Of these, 73 subjects (22 males, aged 79 ± 5 years) had no clinical signs of AD dementia, referred to as the *elderly cohort*, and 28 patients (16 males, aged 70 ± 9 years) had clinically diagnosed disease, referred to as the *clinical AD cohort*. All participants were examined in the Macquarie University Eye Clinic, where a complete medical history was taken and an eye examination was performed, including visual acuity, slit-lamp examination and intra-ocular pressure (IOP) with Goldmann tonometry. Tropicamide 1% was instilled to dilate pupils. The RNFL and retinal ganglion cell layer (RGCL) thicknesses were measured using spectral domain optical coherence tomography (OCT) (Nidek Co., Gamagori, Japan). The amplitudes of retinal vascular pulsations were measured using the Dynamic Vessel Analysis system (Imedos, Jena Germany). Following the ocular tests, all participants visited the Macquarie Heart Clinic (Sydney, Australia) for blood pressure and PWV measurements.

### Elderly cohort

Seventy-three participants were residents from the Anglican Retirement Villages (ARV) across three locations in Sydney. These individuals were participants in the Kerr Anglican Retirement Villages Initiative in Ageing Health (KARVIAH) study, which required them to undertake Aβ-PET imaging. Participants who had reported any subjective memory complaints but were cognitively healthy with no confirmation of dementia by objective neuropsychological assessments were recruited. Inclusion criteria for the study included (1) aged 65–90 years, with good health and no significant cerebrovascular disease; (2) living in independent living units or similar accommodations; (3) English speaker; (4) adequate vision and hearing to enable testing; (5) memory complaint as determined by Memory Complaint Questionnaire and preferably corroborated by an informant; (6) no objective memory impairment (based on cognitive test scores); (7) normal general cognitive function as determined by a Montreal Cognitive Assessment score greater than or equal to 26 during screening; (8) no significant functional impairments/behaviour problems as indicated by the Informant Questionnaire on Cognitive Decline in the Elderly and the 36-item Short Form Health Survey; and (9) intact activities of daily living (ADL) (i.e., no or minimal impairment in ADLs as determined by a clinical interview). Of the 73 participants, 8 (11%) had glaucoma, 5 (7%) had macular degeneration, 41 (56%) had systemic hypertension and were on monotherapy, 7 (10%) had non-insulin-dependent diabetes, 6 (8%) had a history of transient ischaemic attack (TIA) but not confirmed stroke, 5 (7%) had ischaemic heart disease (IHD) and 22 (30%) were past smokers. The subjects visited Macquarie Medical Imaging for neuroimaging, including PET and MRI scans. Within 6 weeks of undergoing imaging, the subjects returned to Macquarie Eye Clinic for their ocular examinations.

### Clinical AD cohort

A further 28 participants diagnosed with AD were referred by Macquarie Neurology. The clinical AD cohort had inclusion/exclusion criteria similar to those of the elderly cohort, and they all underwent the neuropsychological battery used in the Australian Imaging, Biomarkers and Lifestyle (AIBL) study of ageing to confirm clinical AD [18].

### Optical coherence tomography and vascular imaging

All participants underwent retinal OCT. The scans included a peri-papillary RNFL scan and a 9-mm × 9-mm macular scan to determine RGCL complex.

The technique used for measuring retinal vascular pulsation and dynamic vascular response has been described previously [19]. In brief, a digital camera was used to capture retinal images during a short video recording. The recording duration was 180 seconds during which three sets of flicker-induced light stimulated the retina, with each cycle lasting 30 seconds. The retinal image is registered by the software, and video analysis can then be performed, minimising effects of eye movement. Individual arteries and veins at four quadrant locations near the nerve were selected for analysis, and the vascular dilatory response of the vessels in response to flicker was recorded. The amplitude of vessel pulsatility was also determined for both arteries and veins.

### Systemic vascular measurements

The participants were seated, and a standard cuff was placed around the upper arm over the brachial artery to measure blood pressure. Two consistent measurements with no more than 5-mmHg difference between the systolic values were taken and recorded. The subjects then lay supine for carotid-to-femoral PWV measurements to assess peripheral arterial stiffness. A cuff was placed around the femoral artery, and a tonometer sensor was positioned on the carotid pulse. Distances were measured from the cuff to the femoral artery, the cuff to the sternal notch, and the carotid artery to the sternal notch to calculate the time difference from the carotid artery to the femoral artery using the SphygmoCor system (AtCor Medical, West Ryde, Australia).

### Neuroimaging

MRI consisted of three-dimensional, T1-weighted, magnetisation-prepared rapid gradient echo scans (repetition time 2.3 milliseconds, echo time 2.98 milliseconds and flip angle 9 degrees). Following MRI, radiolabelled ^18^F-florbetaben was used for the PET amyloid scans. Participants were administered 1.6 ± 1.2 μg of florbetaben with a specific activity of 105 ± 74 GBq/μmol and radiochemical purity higher than 95%. PET amyloid imaging was then performed. A transmission scan using a rotating ^137^Cs source was obtained for attenuation correction immediately before the emission scan.

### Data analysis

Retinal and florbetaben-PET analyses were performed blinded to each other and to other study data. We processed the PET-FBB images and quantified the retention by the standardised uptake value ratio (SUVR), which is the standardised uptake value normalised by the average uptake in the cerebellum as a reference. The SUVR of the neocortex was computed as the average SUVR in the grey matter masked neocortical region composed of the frontal, superior parietal, lateral temporal, occipital, and anterior and posterior cingulate regions using Computational Analysis of PET from AIBL image-processing software(CapAIBL; https://capaibl-milxcloud.csiro.au/). For further analysis, subjects with a cut-off value greater than 1.4 were then categorised as the preclinical AD group, and participants with a cut-off less than 1.4 were designated as the elderly control group [20]. Neocortical Aβ was expressed as the average SUVR for the regions of interest (ROIs). The ROIs included frontal, superior parietal, lateral temporal, lateral occipital, and anterior and posterior cingulate regions. For a within-group analysis, all measured parameters were assessed for the preclinical group (neocortical amyloid load [NAL] ≥1.4) and the elderly control group (NAL <1.4). The vascular imaging data were analysed by extracting a 20-second window of recording with the highest signal-to-noise ratio selected and then passing it through a low-pass filter with a cut-off frequency of 3 Hz. Peaks and troughs of the signal were detected using spike software (Cambridge Electronic Design, UK) [19], and the difference between these was designated as the pulse amplitude. RNFL, macular thickness and pulsatility of vessels were compared with the SUVR values using analysis of variance (ANOVA) and post hoc analysis between high and low NAL values. Linear regression was then applied to assess the relationships between the vascular imaging and OCT measurements across all subjects, as well as the systemic measurements. Adjustments were made for age, where necessary, before analysis. Prism software (GraphPad Software, La Jolla, CA, USA) was used for all statistical analyses.

## Results

Participant demographics are shown in Table 1. All cohorts had similar characteristics, except for a much higher proportion of females in the elderly and preclinical AD groups. Adjustments for age did not statistically alter the end results.

**Table 1:** Participant demographics

	Elderly control group (NAL <1.4)	Preclinical AD group (NAL ≥1.4)	Clinical AD cohort
Number of participants	50	23	28
Males/females	14/36	9/14	16/12
Mean age, years	79 ± 5	80 ± 4	70 ± 9
Mean IOP, mmHg	14 ± 3	13 ± 3	13 ± 3
Mean SBP, mmHg	153 ± 14	142 ± 18	137 ± 17
Mean DBP, mmHg	83 ± 15	77 ± 18	80 ± 8
Mean Aβ, SUVR	1.17 ± 0.09	1.75 ± 0.24	–

*Abbreviations*: *A*β Amyloid-β, *AD* Alzheimer’s disease, *DBP* Diastolic blood pressure, *IOP* Intra-ocular pressure, *NAL* Neocortical amyloid load, *SBP* Systolic blood pressure, *SUVR* Standardised uptake value ratio

### Retinal vascular parameters

**Fig. 1:**
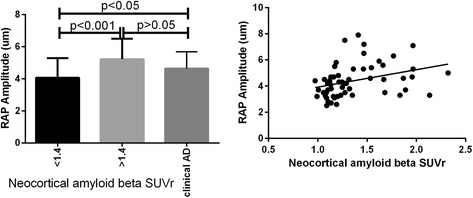
Association between retinal arterial pulsations (RAP) and standardised uptake value ratio (SUVR). *Left*: Mean and SD of RAP across control subjects, preclinical Alzheimer’s disease (AD) and clinical AD. *Right*: Linear association between RAP and SUVR obtained from control subjects and participants with preclinical AD

The amplitude of retinal arterial pulsations (RAP) was assessed and analysed across the three subgroups: (1) participants with an NAL less than 1.4 (*n* = 50), the elderly control group; (2) participants with an NAL greater than 1.4 (*n* = 23), the preclinical AD group; and (3) the clinical AD group (*n* = 28), with the assumption that this group would have an Aβ SUVR greater than 2 [20] (assuming that the diagnosis was 100% accurate, though in clinical practice this figure is around 80%). The mean RAP values across these three groups were 4 ± 1.2 μm, 5.2 ± 1.2 μm and 4.6 ± 1 μm, respectively. One-way ANOVA revealed a significant difference across the three groups (*p* = 0.001). Post hoc multiple comparisons revealed that there were significant differences between groups 1 and 3 (*p* = 0.03) and between groups 1 and 2 (*p* = 0.006), but no significant difference between groups 2 and 3 (*p* = 0.14) (Fig. 1a). The association between RAP amplitude and neocortical Aβ SUVR in the grouped control and preclinical AD cohort was assessed using linear regression. A positive association between the two was observed (*p* = 0.01, *r* = 0.33) (Fig. 1b). RAP amplitude was significantly higher in the preclinical group than in the elderly control subjects. When we added the data from the clinical AD group, the trend did not continue. However, the difference in RAP between preclinical and clinical AD was non-significant. Also, we expect that the RAP trend might have continued to increase if we had the actual Aβ SUVR for this group.

**Fig. 2:**
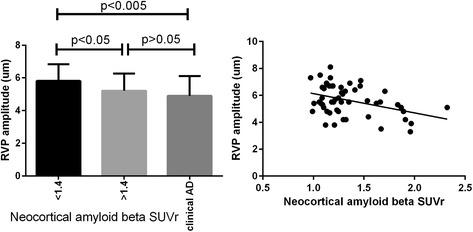
Association between retinal venous pulsations (RVP) and standardised uptake value ratio (SUVR). *Left*: Mean and SD of RVP across control subjects, preclinical Alzheimer’s disease (AD) and clinical AD. *Right*: Linear association between RVP and SUVR obtained from control subjects and participants with preclinical AD

A similar approach was taken when we analysed the amplitude of retinal venous pulsations (RVP). The mean RVP values across these three groups were 5.8 ± 1 μm, 5.2 ± 1 μm and 4.9 ± 1.2 μm, respectively. One-way ANOVA revealed a significant difference across the three groups (*p* = 0.004). Post hoc multiple comparisons revealed that there were significant differences between groups 1 and 3 (*p* = 0.006) and between groups 1 and 2 (*p* = 0.03), but no significant difference was observed between groups 2 and 3 (*p* = 0.37) (Fig. 2a). The association between amplitude of RVP and neocortical Aβ SUVR in the grouped control and preclinical AD cohort was assessed using linear regression. A negative association between the two was observed (*p* = 0.003, *r* = 0.4) (Fig. 2b).

### Flicker-induced retinal vasodilation

We also assessed flicker light-induced retinal vasodilation. Table 2 shows the mean vasodilation percentages in the control (NAL <1.4), preclinical (NAL ≥1.4) and clinical AD cohorts. Although the mean retinal arterial and venous dilation was slightly lower in the preclinical group than in the control subjects, we did not observe any statistically significant difference (*p* = 0.39). The relationship between neocortical Aβ SUVR and retinal arterial and venous dilation, assessed by linear regression, was also non-significant (*p* = 0.1).

**Table 2:** Flicker-induced retinal vasodilation

	Arterial dilation (%)	Venous dilation (%)
Elderly control group (NAL <1.4)	2.4 ± 1	3.1 ± 1.2
Preclinical group (NAL ≥1.4)	2 ± 1.1	3 ± 1.5
Clinical AD	2.7 ± 0.5	2.8 ± 1

*AD* Alzheimer’s disease, *NAL* Neocortical amyloid load

### Retinal structural measurements

**Fig. 3:**
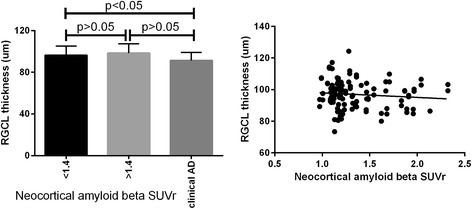
Association between retinal ganglion cell layer (RGCL) thickness and standardised uptake value ratio (SUVR). *Left*: Mean and SD of RGCL thickness across control subjects, participants with preclinical Alzheimer’s disease (AD) and participants with clinical AD. *Right* Linear association between RGCL thickness and SUVR obtained from control subjects and participants with preclinical AD

The average RNFL thickness across the three groups (i.e., control subjects [NAL <1.4], preclinical AD [NAL ≥1.4] and clinical AD) was 93 ± 18 μm, 101 ± 15 μm and 99 ± 17 μm, respectively. We did not observe a significant difference in RNFL thickness between the three groups (*p* > 0.05). The association between the amplitude of RNFL thickness and neocortical Aβ SUVR in the grouped control and preclinical cohort was also not significant (*p* > 0.05). The average RGCL thicknesses across the three groups were 96 ± 9 μm, 98 ± 9 μm and 91 ± 2 μm, respectively. A significant difference in RGCL thickness across the three groups was observed (*p* = 0.02). Post hoc multiple comparisons revealed that there were significant differences between groups 1 and 3 (*p* = 0.03) and between groups 2 and 3 (*p* = 0.005), but no significant difference was observed between groups 1 and 2 (*p* = 0.7) (Fig. 3a). The association between RGCL thickness and neocortical Aβ SUVR in the grouped control and preclinical cohort, as assessed by linear regression, was also not significant (*p* = 0.27). Collectively, there was only a small degree of retinal structural changes identifiable with OCT, and this was found only in the RGCL analysis.

### Systemic vascular parameters

We also investigated for differences in systemic cardiovascular parameters between the subgroups. Pulse pressure (PP = systolic blood pressure − diastolic blood pressure) and PWV were assessed. The average PP and PWV across the three groups were, respectively, 68 ± 13 mmHg, 8.8 ± 1.4 m/second; 65 ± 12 mmHg, 8.7 ± 1.5 m/second; and 67.4 ± 13.3 mmHg, 8.8 ± 1.4 m/second. There was no significant association between PP, PWV and neocortical Aβ SUVR in the grouped control and preclinical cohort (*p* = 0.4) across all subjects. Subgroup analysis also did not reveal any significant difference (*p* = 0.3) between PP, PWV and neocortical Aβ SUVR in the elderly subjects vs the preclinical AD group.

Finally, we applied an analysis of covariance to investigate whether co-morbidities including hypertension, diabetes, TIA, IHD and a history of smoking influenced the association between RVP, RAP and Aβ SUVR. The results showed that the differences between the slopes were not significant (*p* > 0.05).

## Discussion

In this study, we investigated the association between retinal haemodynamics and cerebral Aβ load in a cohort of elderly participants with no clinical diagnosis of AD but who had subjective memory complaints, and we subdivided these for further analysis according to PET scan results (elderly control subjects [NAL <1.4], preclinical AD [NAL ≥1.4]). Participants with confirmed clinical signs of AD were also included to replicate the trend observed in our preclinical AD cohort. We observed a negative correlation between amplitude of RVP and Aβ SUVR in the preclinical cohort. The correlation was positive when we compared RAP amplitude and Aβ SUVR. When data from our clinical AD cohort were added for comparison, with the assumption that these patients would have an Aβ SUVR greater than 2.0 [20], we observed a continuing trend in our control subjects and preclinical AD cohort, further supporting the association between retinal haemodynamics and cerebral Aβ load.

Recent studies have shown a significant association between measures of vascular function (PP, arterial stiffness, endothelial function, carotid intima-media thickness, endothelial cell response to stress) and cognitive function [16, 21, 22]. Mechanical forces on endothelial cells result in activation of a range of cell-signalling pathways as well as alterations in cell morphology, cell function and gene expression [23]. In contrast, effects of dynamic forces due to pulsatile pressure and flow result in cyclic strain on the cell membrane. Cyclic stretch has also been shown to induce retinal expression of vascular endothelial growth factor and has been implicated in the exacerbation of diabetic retinopathy by high blood pressure [24].

Recent studies have highlighted the link between endothelial nitric oxide (NO) and cerebrovascular function, in modulation of the processing of APP, in affecting the functional status of microglia, and on cognitive function [25]. Loss of NO in cultured human cerebrovascular endothelium has been shown to increase the expression of APP and β-site amyloid precursor protein-cleaving enzyme 1 (BACE1), thereby resulting in increased secretion of Aβ peptides (Aβ_1–40_ and Aβ_1–42_) [26]. Increased expression of APP and BACE1, as well as increased production of Aβ peptides, was also detected in the cerebral microvasculature and brain tissue of endothelial nitric oxide synthase-deficient mice.

Collectively, the higher retinal arterial pulse amplitude observed may be due to mechanical cyclic stretch (to simulate the mechanical pulsatile effect of arterial pressure) in response to upregulation of APP expression as well as deposition of Aβ plaques. Further studies are required to elucidate underlying mechanisms involved in regulating retinal arterial pulse amplitude in AD. It may be a primary risk factor, or it may occur secondary to local endothelially induced changes.

With respect to static retinal imaging, many researchers have investigated the correlation between retinal vascular calibre and cognitive decline [27–29]. However, the limitation with static measures is that often the biomarkers observed are not specific to the disease and can be seen in other diseases that may affect retinal pathology (e.g., hypertension, diabetes). In the Rotterdam study, researchers reported that *larger* retinal venular calibres were associated with an increased risk of dementia [30]. These findings are contradictory to those of another study in which investigators reported that *narrower* venular calibre is associated with likeliness of AD development [28]. In our cohort, we did not observe any significant association between retinal venous and arterial diameters with Aβ SUVR. However, studying dynamic indices of retinal vessels, including retinal pulsatility and local arterial PWV, within retinal vessels (a measure of arterial stiffness) is more likely to be applicable in identifying those at risk.

In this study, we also investigated endothelial dysfunction in retinal vasculature and its association with neocortical Aβ scores. Endothelial dysfunction plays a significant role in the pathogenesis of vascular abnormalities, and various feedback mechanisms are involved in retinal autoregulation. Previous studies have demonstrated that NO is involved in retinal vasodilation during flicker light stimulation [31, 32]. Although we observed significantly lower vasodilation in retinal arteries and veins in the clinical AD cohort in comparison with the participants having an Aβ SUVR less than 1.4, we did not see any significant association between the percentage of retinal vasodilation during flicker stimulation and Aβ SUVR.

The origin of spontaneous RVP in the eye is debated, with some researchers suggesting abnormalities in the ocular perfusion pressure in combination with high central retinal vein pressures as the main factor [33–35], whereas others argue for an imbalance in translaminar pressure gradient across the lamina cribrosa (IOP-CSF pressure) [36, 37]. Lower venous pulsations are also observed in primary open angle glaucoma [38]. Glaucoma and AD share common features of age-related risk, chronic decline in function and focal degeneration of neurons. Both diseases are characterised as degenerative disorders involving the CNS, and even though glaucoma is primarily an optic nerve disorder, secondary changes have been observed in the radiations and visual cortex. Glaucoma has been reported to be associated with AD pathophysiology [39, 40]. There are also reports suggesting that glaucoma prevalence is higher in AD (2.6 to 5.2 times) [41]; both diseases are associated with microbleeding [42, 43]. In our cohort, we did find a higher-than-average incidence of subjects with glaucoma (8 of 73 [about 11%]), but subanalysis did not identify this group of participants as showing any significant difference in parameters other than thinner RGCL/RNFL, as expected.

Retinal structural changes, including RNFL and RGCL thickness, have been reported in AD [44–46]. Although most reports of thinning of RNFL and GCL are associated with AD severity, some suggest a non-significant correlation between retinal structures and AD severity [45, 47]. Our findings show a non-significant correlation between RNFL thickness and Aβ SUVR. When assessing macular RGCL, we observed only a slightly significant reduction in the thickness when we compared patients with clinical AD with the elderly control subjects and participants with preclinical AD. The results were not changed when the subjects with glaucoma and macular degeneration were excluded.

The vascular contribution to the broad range of cognitive impairment leading to AD has been recognised [48, 49], including by professional societies providing statements based on epidemiological evidence linking loss of vascular function and development of dementia [50]. Studies of associations of vascular function and cognitive impairment are converging on the question whether AD can be considered part of the spectrum of vascular disease [51]. There is accumulating evidence suggesting an association of arterial blood pressure, arterial stiffness and reduced endothelial function with AD [22, 52], but there have been conflicting findings in the literature, and it is clearly not a simple relationship with arterial blood pressure per se. Infusing hypertensive doses of angiotensin II in *APP*/*PS1*-transgenic mice produced a 100% increase in soluble Aβ in the brain and a 25% reduction in cerebral microvessel density [53]. We assessed central PP (systolic − diastolic) and PWV and their association with Aβ SUVR. No significant association or correlation was observed between these cardiovascular parameters and neocortical Aβ scores.

Limitations of our study include that we did not have neuroimaging data from our clinically confirmed AD group, because we could not ethically justify scanning these patients. It was anticipated that 80% of participants with clinical AD would have high PET scores (>2). Additionally, there might be a false-positive rate within our cohort (i.e., healthy elderly subjects with SUVR >1.4) leading to classification as preclinical AD. Whereas this would not affect the association between SUVR and retinal vascular changes, future scans and recording will elucidate the number of false-positives within the cohort. We also acknowledge that a large proportion of our cognitively healthy participants were receiving treatment for systemic hypertension. In this age group, this is extremely common, but if our hypothesis is correct that there is a link with arterial pulse waves and chronic brain damage, then this treatment may be most likely masking the effect.

## Conclusions

This study demonstrates a significant correlation between amplitude of retinal vascular pulsatility and neocortical Aβ scores, independent of other risk factors. Arterial pulsation was increased while venous pulsation was reduced. Future studies will further elucidate whether these biomarkers have a correlation not only with cerebral Aβ plaques but also with the development of subsequent clinical dementia, because these participants are all being followed longitudinally. If a clinical correlation is confirmed, although direct cause and effect cannot be certain, screening of eyes in those considered at risk of AD may aid in the development of an additional inexpensive approach for identifying increased risk as well as serving to potentially monitor vascular function in these individuals.

## Abbreviations

AD: Alzheimer’s disease
ADL: Activities of daily living
AIBL: Australian Imaging, Biomarkers and Lifestyle study
ANOVA: Analysis of variance
APP: Amyloid precursor protein
ARV: Anglican Retirement Villages
Aβ: Amyloid-β
BACE1: β-Site amyloid precursor protein-cleaving enzyme 1
CNS: Central nervous system
CSF: Cerebrospinal fluid
DBP: Diastolic blood pressure
IHD: Ischaemic heart disease
IOP: Intra-ocular pressure
KARVIAH: Kerr Anglican Retirement Villages Initiative in Ageing Health study
MRI: Magnetic resonance imaging
NAL: Neocortical amyloid load
NO: Nitric oxide
OCT: Optical coherence tomography
PET: Positron emission tomographic
PP: Pulse pressure
PWV: Pulse wave velocity
RAP: Retinal arterial pulsations
RGCL: Retinal ganglion cell layer
RNFL: Retinal nerve fibre layer
ROI: Region of interest
RVP: Retinal venous pulsations
SBP: Systolic blood pressure
SUVR: Standardised uptake value ratio
TIA: Transient ischaemic attack
